# BPA Endocrine Disruptor Detection at the Cutting Edge: FPIA and ELISA Immunoassays

**DOI:** 10.3390/bios13060664

**Published:** 2023-06-19

**Authors:** Anna Raysyan, Sandro D. Zwigart, Sergei A. Eremin, Rudolf J. Schneider

**Affiliations:** 1 BAM Federal Institute for Materials Research and Testing, 12205 Berlin, Germany; 2 Department of Chemistry, Humboldt-Universität zu Berlin, 12489 Berlin, Germany; 3 Faculty III Process Sciences, Technische Universität Berlin, 10623 Berlin, Germany; 4 Chemical Faculty, M.V. Lomonosov Moscow State University, Moscow 119991, Russia

**Keywords:** FPIA, ELISA, immunoassay, bisphenol A, endocrine disruptor

## Abstract

BPA is a chemical commonly used in the production of polymer-based materials that can have detrimental effects on the thyroid gland and impact human reproductive health. Various expensive methods, such as liquid and gas chromatography, have been suggested for detecting BPA. The fluorescence polarization immunoassay (FPIA) is an inexpensive and efficient homogeneous mix-and-read method that allows for high-throughput screening. FPIA offers high specificity and sensitivity and can be carried out in a single phase within a timeframe of 20–30 min. In this study, new tracer molecules were designed that linked the fluorescein fluorophore with and without a spacer to the bisphenol A moiety. To assess the influence of the C6 spacer on the sensitivity of an assay based on the respective antibody, hapten–protein conjugates were synthesized and assessed for performance in an ELISA setup, and this resulted in a highly sensitive assay with a detection limit of 0.05 g/L. The lowest limit of detection was reached by employing the spacer derivate in the FPIA and was 1.0 μg/L, working range from 2 to 155 μg/L. The validation of the methods was conducted using actual samples compared to LC–MS/MS, which served as the reference method. The FPIA and ELISA both demonstrated satisfactory concordance.

## 1. Introduction

Human exposure to bisphenol A (BPA) has increased as its use has increased globally. BPA is a monomer that is used in the manufacturing of polycarbonate plastic products as well as epoxy resins that line metal cans, both of which come into contact with food and beverages [1]. Excessive exposure to BPA, which is an endocrine disruptor, could impact human reproductive health. To address this issue, extensive research has been conducted on its effects on animals and humans. As a result, various countries have taken measures, from voluntary reductions to complete bans on BPA in certain products, such as baby bottles and canned food containers. Many methods have been developed for the detection of BPA, including instrument-based methods such as high-performance liquid chromatography and gas chromatography, mostly coupled to tandem mass spectrometry (LC–MS/MS, GC–MS/MS) [2,3,4,5,6,7]. The main drawbacks of these methods are their high cost, long analysis time, and difficulty in analyzing a large number of samples. Despite these challenges, consumers would welcome a fast test to detect contamination by bisphenol A. Compared to instrument-based methods, immunoassays have advantages such as simplicity, specificity, low chemicals consumption, and high sensitivity, making them a desirable choice for rapid detection [8,9]. Various sensor platforms, particularly electrochemical ones, have shown successful analytical performance using aptamers. However, the lack of diversity in aptamers used for BPA detection is a limitation. New aptamers with higher selectivity could improve detection. Different signal amplification strategies have been explored, but cost and current limit values influence their application. Continuous onsite analysis and miniaturization are crucial. Overall, aptamers hold promise for BPA detection and its analogs [10]. Fluorescence ELISA has gained interest in analyzing pollutants due to its simplicity, specificity, and high throughput. Fluorescence ELISA (FELISA) is an attractive immunoassay that combines immunoassay advantages with fluorescence analysis for high sensitivity. However, FELISA has drawbacks including high background fluorescence and fluorescence quenching due to the aggregation-caused quenching (ACQ) effect. To overcome these limitations, copper nanoclusters (CuNCs) with aggregation-induced emission (AIE) have been used as fluorescence probes due to their intense emission in aggregated states. However, CuNCs suffer from poor stability and low emission. Encapsulating AIE materials into porous structures, such as metal–organic frameworks (MOFs), has been explored to enhance AIE properties and stability [10]. 

Fluorescence polarization immunoassay (FPIA) is a type of homogeneous immunoassay that is quick and easy to perform, taking only 20 to 30 min. The test measures the fluorescence emission of an analyte–fluorophore conjugate (also known as the tracer), which is the source of the signal in the test [11,12,13,14].

**Figure 1:**
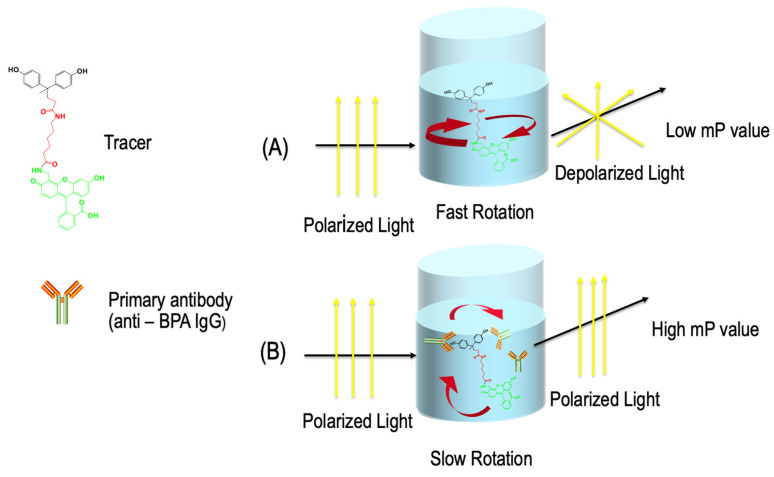
FPIA principle illustration for determining BPA.

The sample is mixed with a reagent and excited using plane-polarized light generated by a polarizer between lamp and sample cuvette. The emitted fluorescence radiation is measured using a photomultiplier behind another polarizer. The intensity of the incident light is recorded as “parallel” (I_║_) and then a second value after rotating the second polarizer by 90° (“perpendicular”, I**_⊥_**). Some instruments have a second detector in a 90° position that records I**_⊥_** simultaneously. The difference between both recordings divided by their sum (assuming equal sensitivity in both directions) is referred to as fluorescence polarization (FP) measured in milliP (Figure 1). When an antibody is added to the solution, the fluorescence polarization value can be used to indicate the concentration of the analyte in the sample. A high analyte concentration results in low polarization, while a low analyte concentration leads to high polarization. This relationship between polarization and analyte concentration is plotted as a sigmoidal curve on a logarithmic scale. Fluorescein is the most commonly used fluorophore, and instruments are calibrated to measure its peak excitation at 494 nm and peak emission at 521 nm. 

The development of a fluorescence polarization immunoassay (FPIA) utilizing specific antibodies against bisphenol A (BPA) will enable rapid and sensitive detection of BPA contamination in various polymer samples, offering a cost-effective alternative to instrument-based methods and time-consuming immune analytical techniques. 

In this study, we designed new tracer molecules for bisphenol A detection. By incorporating a C6 spacer, we achieved high sensitivity in both an ELISA and a fluorescence polarization immunoassay (FPIA). We validated these methods using actual samples compared to LC–MS/MS as the reference, and both the FPIA and ELISA showed good agreement.

The research presented in this article is part of Anna Raysyan’s doctoral dissertation, which concerns immunoassay techniques that are inexpensive, adaptable, fast, and user-friendly for detecting bisphenol A, an endocrine-disrupting compound. The current article focuses specifically on developing a fluorescence polarization immunoassay (FPIA) for BPA determination [15].

## 2. Materials and Methods

### 2.1. Chemicals

Nunc provided transparent microtiter plates with 96 flat-bottom wells and high protein binding capacity (MaxiSorpTM) (Thermo Scientific, Bremen, Germany). Greiner bio-one provided UV-transparent 96-well microtiter plates, UV-StarTM (Frickenhausen, Germany). TLC plates used were Merck KGaA silica gel 60 with/without concentration zone and with/without fluorescence indicator (Darmstadt, Germany). Merck KGaA filter syringes with a TeflonTM membrane, a pore size of 0.45 m, and a diameter of 17 mm (Darmstadt, Germany) were used. GE Healthcare provided PD-10 columns containing Sephadex G-25 (Munich, Germany). 

Professor Chuanlai Xu’s laboratory (School of Food Science & Technology, State Key Lab of Food Science and Technology Jiangnan University, Wuxi, Jiangsu, China) provided anti-bisphenol A mouse monoclonal antibody was provided by the lab of buffered (7 mg/mL in 0.01 M PBS, pH 7.4, 1% BSA, 1% glycerol, 0.02% azide) [15,16]. Polyclonal goat anti-mouse HRP antibody (clone A4416, with 0.5–3 mg/mL in 0.01 M PBS, pH 7.4, 1% BSA, 0.02% azide) was provided by Merck Millipore, (Darmstadt, Germany). 

Merck KGaA (Darmstadt, Germany) provided the N-hydroxysulfosuccinimide (Sulfo-NHS), 2-(N-morpholino) ethanesulfonic acid (MES), 1-ethyl-3-(3-dimethylaminopropyl)carbodiimide*HCl (EDC) bovine serum albumin (BSA), bisphenol A (BPA), bisphenol valeric acid (BVA), bisphenol A-d16 (BPA-d16), bisphenol B (BPB bisphenol F (BPF)), 4-cumylphenol (4-CP),bisphenol E (BPE), bisphenol S (BPS), 4-octylphenol (OCP), and 4-nonylphenol (4-NP). 

4′-(Aminomethyl)fluorescein hydrochloride (AMF) was obtained from Invitrogen (Carlsbad, CA, USA). Methanol (MeOH) and ethanol were obtained from J.T. Baker (Griesheim, Germany). 

### 2.2. Instruments and Equipment

Spectrophotometer SpectraMax Plus384 from Molecular Devices (Ismaning, Germany) at 450 nm and referenced to 620 nm, controlled using SoftMax^®®^ Pro software (v 5.2, Molecular Devices) was used. All ELISA incubation steps were carried out at room temperature on a Titramax 101 plate shaker (Heidolph, Schwabach, Germany) set to 750 rpm. The plates were washed with an automatic 96-channel plate washer (BioTek Instruments, EL^x^405 SelectTM, Bad Friedrichshall, Germany) in between individual incubation steps. A PBS-based washing buffer (0.75 mM potassium dihydrogen phosphate, 6.25 mM dipotassium hydrogen phosphate, 0.025 mM sorbic acid potassium salt, 0.05% (*v*/*v*) TweenTM 20, pH 7.6) was used for three cycles of washing.

A Sentry 2000Si was used to measure fluorescence polarization (Ellie LLC, Wauwatosa, WI, USA). The Sentry 2000Si is a fluorescence polarization instrument with multiple wells. In black 8- or 12-well microplate strips, reactions are read. The instrument is outfitted with a high-volume precision ceramic fluid metering systems pump. 

MALDI-ToF mass spectra were obtained using a Bruker Reflex III MALDI mass spectrometer from Bruker-Daltonik (Bremen, Germany), which was powered using a nitrogen laser and operated at a 20 kV acceleration voltage.

LC–MS/MS was used to determine BPA reference concentrations in samples using an Agilent 1260 Infinity LC system equipped with a binary pump, degasser, autosampler, and column heater. Chromatographic separation was performed on a Kinetex XBC18, 100, 2.6 m, 150 3 mm analytical LC column with a guard column of UHPLC C18, 3 mm (both Phenomenex, Aschaffenburg, Germany). Milli-Q water with 10 mM NH4Ac and 0.1% (*v*/*v*) acetic acid (A) and MeOH with 10 mM NH4Ac and 0.1% (*v*/*v*) AcOH (B) were used as mobile phases. The system was run at of 350 μL min*^−^*^1^ flow rate and 30 °C column heater temperature. For the first 15 min, an elution gradient of 80% A was used. Within 5 min, A was reduced to 5% (95% B). Then, A was ramped up to 80% in 0.5 min and held there for 14.5 min to re-equilibrate the column. A sample volume of fifteen microliters was injected. An ABSciex 6500 Triple Quad mass spectrometer was used for mass spectrometric detection. Positive ionization electrospray ionization (ESI) was used.

### 2.3. Preparation of Hapten–Protein Conjugates

Schmidt et al. [17] and Raysyan et al. [18] found that the way in which conjugated hapten is chemically structured greatly affects its ability to bind to an anti-BPA antibody. They described that a six-carbon linear aliphatic chain was the best spacer for achieving optimal binding. Therefore, to increase the assay’s sensitivity, it is essential to synthesize functional derivatives of the target compound. To achieve this, the researchers coupled 4,4-bis(4-hydroxyphenyl) valeric acid (BVA) with aminohexanoic acid (Ahx). 

Briefly, BVA (26.3 mg) and NHS (12.9 mg) were dissolved using EDC (22.3 mg) in 3 mL DMSO and stirred for 2 h under argon at room temperature in an amber glass vial. A quantity of 1.5 mL DMSO was used to dissolve Ahx (9.8 mg), which was then mixed with 1.5 mL PBS. (pH 6). A quantity of 2.5 mL of the reacted BVA solution was added dropwise to the Ahx solution and incubated at room temperature for 4 h. BVA–Ahx is not indefinitely stable and is most likely decomposed (partially) during preparative LC purification. (Figure S5). 

Therefore, the freshly prepared BVA–Ahx solution was added to the appropriate protein solution and stirred at room temperature for 4 h. Twenty-two milligrams of BSA was dissolved in 2 mL of PBS pH 6. The activated BVA–Ahx was added to the BSA solution dropwise. The protein conjugate was purified using SEC on a PD-10 column after 4 h of reaction time. Hapten–protein concentration of BVA–BSA of 2.1 mg/mL, and for BVA–Ahx–BSA (Figure S1) of 2.48 mg/mL, were determined using the Bradford assay [19]. MALDI-ToF MS was used to confirm the efficiency of the conjugation reaction (Figures S3 and S4). 

### 2.4. Synthesis of Fluorescein-Labeled Tracers (BVA–AMF and BVA–Ahx–AMF)

Eremin and colleagues’ [12,14,20,21,22] NHS-activated ester method, with slight adjustments, was utilized to link the fluorescent tags to the haptens. BVA and BVA–Ahx (Figure S1) NHS activated esters were created beforehand and added to 1.05 mg (2.6 µmol) AMF dissolved in 10 µL of triethylamine. A yellow-orange solution was produced for all products, which was further stirred for 4 h before purification using thin-layer chromatography (TLC) (Table S1).

Silica plates (2.5 × 7.5 cm; silica gel 60 with a concentration zone, devoid of a fluorescence indicator and manufactured by Merck) were utilized to conduct TLC. The mobile phase comprised CHCl_3_:CH_3_OH (4:1, *v/v*). The primary yellow band, easily visible under UV light (λ = 365 nm), was gathered and dissolved in ethanol for each tracer. This solution was then filtered through a Teflon membrane syringe filter, 17 mm in diameter, possessing a pore size of 0.45 µm. The resulting product was then purified again using TLC (refer to retardation factors Rf in Table S1). The ethanol solvent was evaporated, and the residue was dissolved in 100 µL methanol. The solution was stored at 4 °C and later used directly as a tracer stock solution for creating dilutions (Tracer Working Solutions) in borate buffer.

Confirmation of successful synthesis was achieved using LC–MS/MS analysis (refer to Figures S6–S8). Mass spectra were obtained at the retention time of the primary peak in the UV trace. The *m*/*z* + 1 and +Na+ adduct ions were detectable for each specific product of the synthesis. Although some compounds exhibited traces of impurities, as evidenced by the chromatograms, the majority of the compounds showed high purity.

### 2.5. Protocol of the Indirect ELISA

The checkerboard titration method was employed to establish the optimal dilutions of BVA–BSA or BVA–Ahx–BSA and anti-BPA mouse IgG [15,18]. To coat the microtiter plates with the corresponding conjugate, 200 µL per well of PBS pH 7.5 was utilized. The coated plates were then sealed using Parafilm^®^ and incubated overnight for approximately 18 h on a plate shaker at 750 rpm. The microtiter plates utilized were of the high-binding, transparent variety. Following the incubation period, the plates were rinsed thrice with washing buffer. Following the washing step, the plates were blocked using casein in PBS (1%, *w/v*, 200 μL per well) for 1 h. After washing the plates, 100 µL of either the sample or BPA standards, along with diluted anti-BPA monoclonal IgG, was added to each well. The plates were then incubated for a duration of 1 h. Following the three-cycle washing step, 100 μL HRP-conjugated goat anti-mouse IgG diluted 1: 20,000 in PBS buffer was added in each well and incubated for 1 h. After additional washing step, 200 μL substrate solution (TMB) was added. For a single plate, 22 mL citrate buffer pH 4.0 with 8.5 μL H_2_O_2_ (30%) and 550 μL TMB solution (40 mM TMB, 8 mM tetrabutylammonium borohydride, in N,N′-dimethylacetamide) were prepared and 200 μL of this mixture was added to each well of the microtiter plate. The plate was then incubated for a period of 20 min, halted by the addition of 100 μL H_2_SO_4_ (1 M). Photometric measurements of absorbance were recorded using a SpectraMax Plus384 spectrophotometer from Molecular Devices, which was controlled using SoftMax^®^ Pro software. The measurements were obtained at a wavelength of 450 nm, with reference to 620 nm.

### 2.6. Protocol of the FPIA

To construct the FPIA calibration curve, a total of 120 µL of borate buffer, 40 µL of Milli-Q water (for the blank) or standard solution, and 30 µL of the tracer working solution (TWS) were added to each microwell of an 8-well strip. Following this, the blank value (mP0) was recorded after a specific tracer incubation time. Subsequently, 30 µL of an antibody solution was added to each well. The mixture was then incubated for an optimal period of time for antibody binding, and the mP value was determined [14].

FPIA is a kinetic assay that exhibits a time-dependent change in degree of polarization, and full equilibrium is not reached within the desired short incubation period. Therefore, the tracer and antibody incubation times must be separately evaluated in order to optimize the assay. In addition, the assay requires mixing and shaking for reproducibility (as depicted in Figure S2).

The mP of the blank (mP0) was determined first, and then each mP value read was divided by mP0. To calibrate, the results were plotted against the logarithm of the BPA concentration, and a sigmoidal curve described by a logistic, four-parameter equation was assigned to the data points using Origin 8G Software. (OriginLab, Northampton, MA, USA).

### 2.7. Sample Preparation

Several plastic samples (Table 1 were collected, and 1 g was thoroughly mixed for 3 min with 30 mL of dichloromethane before being placed in a conical flask in an ultrasonic bath at 25 °C for 45 min. Afterwards, 70 mL of methanol was added (dropwise until precipitate of the polymer forms), and the sample was then filtered through a 0.45 µm filter and diluted in methanol for the LC–MS/MS and in MilliQ water for the FPIA and ELISA [15,18].

## 3. Results

### 3.1. Characterization of the Hapten–Protein Conjugates

A well-designed hapten structure is essential for developing highly sensitive and specific immunoassays. There are two methods for designing and synthesizing BPA haptens. The first approach, known as the homologous approach, aims to preserve the two phenolic groups as potentially immunodominant epitopes. The second approach, called the heterologous approach, is based on the idea that in a competitive format, sensitivity can be optimized through boosting the antibody’s affinity for the analyte over the hapten conjugate competitor, which differs in structure more than in the homologous format [17]. 

Commercial bisphenol valeric acid (BVA) was used as a hapten in the first approach. The carboxyl group of BVA was utilized for the conjugation of BSA protein, similar to the immunogen, while the two phenol groups were left available for immunorecognition. The resulting antibodies were anticipated to specifically recognize these two phenolic groups in the hapten [15]. The second method involved using 6-aminohexanoic acid (Ahx) as a linker with six carbons as a spacer, which was a novel approach in synthesizing a BVA hapten–protein conjugate. This approach was adopted to increase flexibility and generate a higher affinity differentiation between the analyte and a competing hapten using the antibody. The outcome of this study showed that the coupling of haptens to BSA was successful.

The measurement range was assessed by applying the concept of the precision profile. A function, $y=\left(a\times x^{b}\right)+c+\left(\frac{d}{x^{e}}\right),$ developed before [23,24,25], allows for the fitting of a continuous line to the data points of the precision profile with *a*, *b*, *c*, *d*, and *e* being variables and *x* the concentration of the analyte. 

**Figure 2:**
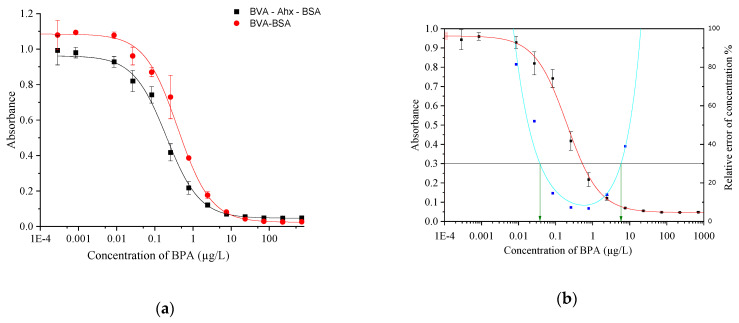
(**a**) Calibration curves obtained using the two conjugates (BVA–BSA and BVA–Ahx–BSA). Table S2 demonstrates the four-parameter fitting parameters. (**b**) ELISA calibration curve with conjugate BVA–Ahx–BSA (red solid line), measurement range (indicated by green arrows) from 0.05 to 8 μg/L (intersection points at 30% relative error of concentration, solid black lines), precision profile (blue squares and cyan line). A relative error of the determined concentration of 30% was considered acceptable to mark the limits of detection (LOD). A detection range of over two decades was established with a lower detection limit of 0.05 µg/L and an upper detection limit of 8 µg/L.

Following conjugate preparation, the indirect competitive ELISA was optimized using checkerboard titrations. The ideal concentration of the primary antibody for BVA–BSA was found to be 9 ng/mL, and for BVA–Ahx–BSA it was 7 ng/mL. The dilution for BVA–BSA was 1:550,000, and for BVA–Ahx–BSA it was 1:600,000. The optimal dilution for goat anti-mouse HRP was 1:20,000. According to the graph in Figure 2a, this indicates that the BVA–Ahx–BSA conjugate produced a lower IC_50_ value, implying a higher sensitivity. The results demonstrate that the hapten–protein conjugate, which included a C6 spacer, exhibited satisfactory precision and sensitivity with an IC_50_ of 0.2 µg/L. A lower IC_50_ value corresponds to greater sensitivity, thereby allowing for more precise measurements.

Our research findings indicate that using the IgG pair with the BVA–Ahx–BSA system results in improved sensitivity compared to the BVA–BSA system, as presented in Table 2 and Figure 2a,b. Consequently, we opted to use the BVA–Ahx–BSA conjugate for future assay performance characterization and validation. To determine the limits of the measurement range and reliably calculate the value of the LOD, we plotted the precision profile [15,17,18] for the assay using the BVA–Ahx–BSA conjugate, which is shown in Figure 2b.

### 3.2. FPIA: Selection of Optimal Tracer/Antibody Characterization

The antibody/tracer combination is critical for the sensitivity, reproducibility, selectivity, and reliability of FPIA and should always be thoroughly examined. The aim of this study was to investigate the impact of tracer structure on FPIA assay sensitivity by synthesizing new tracers. This was achieved by utilizing the amino group in AMF and coupling BVA via its carboxylic acid group. In order to compare the effectiveness of this approach, 6-aminohexanoic acid (6-Ahx) was used as a spacer in two instances.

In our FPIA setup, the intensity of the borate buffer without the tracer is about 37,000 in both orientations, and the fluorescence polarization is close to zero. Adding a tracer dilution of 1:10,000 results in a 5-fold increase in intensity, reaching about 200,000. This increase is necessary for stable readings on the instrument and can be achieved by increasing the tracer concentration. The optimal tracer incubation time must be determined by observing the signal development and waiting for 2.5 to 6 min before adding the antibody. Tracers made from fluorescein retain their immunoreactivity and high quantum yield, but the working dilutions must be freshly prepared every day as they are unstable to elevated temperatures, pH changes, and light exposure [14,20,21].

The addition of the antibody and subsequent determination of mP reveals that a tracer that effectively binds to the antibody results in a significant decrease in mP as the antibody is diluted, following a sigmoidal pattern (as depicted in Figure S2). Conversely, if the tracer fails to bind to the antibody, there is no noticeable change observed as the antibody is diluted. The antibody dilution curves shown in Figure S2 demonstrate that both synthesized tracers effectively bind to the antibody. The optimal dilution of the antibody can be determined from these curves, which correspond to a signal change of approximately 50% of the maximum (as detailed in Table 1). Dilutions higher than this yield negligible signal changes, while dilutions lower than this affect the antibody. The recommended dilutions fall within the range of 1:100 to 1:20,000.

**Figure 3:**
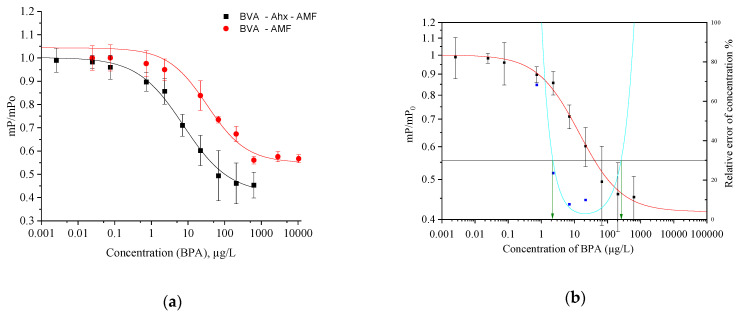
(**a**) The calibration curves for the two optimized tracers (BVA–AMF and BVA–Ahx–AMF) are shown, along with the parameters of the four-parameter fitting, as detailed in Table S3. (**b**) The FPIA calibration curve is illustrated using the conjugate BVA–Ahx–AMF (represented by a red solid line) alongside the precision profile (depicted by blue squares and a cyan line) and measurement range (indicated by green arrows). The measurement range was determined using intersection points at a 30% allowed relative error of the determined concentration (as shown by the black line) and spans from 2 to 155 μg/L.

The structure of the tracer can have a significant impact on the performance of the immunoassay, as indicated in Table S3. The spacer Ahx (C6) plays a crucial role in the binding reaction, with higher sensitivity observed when using the BVA–Ahx–AMF tracer (IC_50_, 7.5 µg/L) and BVA–AMF (IC_50_, 33.4 µg/L) (Figure 3a,b). The optimal tracer was selected based on the IC_50_ and LOD parameters of the assay. This phenomenon has also been observed in other studies [25,26,27]. As a result, the tracer BVA–Ahx–AMF was ultimately chosen for further investigations (as illustrated in Figure 3b).

### 3.3. Selectivity of Antibody

Cross-reactivity is an important factor in the specificity of any immunoassay, including FPIA. In the case of BPA, analogues with similar structures showed varying levels of cross-reactivity depending on the method used. For instance, some analogues may have a higher degree of cross-reactivity in ELISA, while others may have a higher degree of cross-reactivity in FPIA. This variability highlights the importance of considering cross-reactivity in the selection and validation of immunoassays for specific analytes [18]. 

Several compounds structurally related to bisphenol A were tested for immunoassay selectivity and monoclonal antibody specificity (Table S4). This was assessed by calculating their cross-reactivity (CR, in%) and determining their IC_50_ values as follows:

$${CR}_{\%}=\frac{IC_{50}\left(BPA\right)}{{IC}_{50}\left(test\;compound\right)}\times 100\%$$

In the case of BVA, 4-CP, BPE, BPF, and BPS molecules, demethylation and removal of the bisphenol A hydroxyl groups resulted in a significant reduction in cross-reactivity. Furthermore, bisphenol B (BPB), with an additional carbon, resulted in a cross-reactivity of approximately 200%. This very high level of cross-reactivity for this specific compound can be specified by the higher similarity of the compound to the antigen used for immunization. The antibody was created using a BVA hapten–protein conjugate. 

Nevertheless, bisphenol A analogues that are more intricate, such as bisphenol S (BPS), were unable to be detected. Simple phenolic compounds with low cross-reactivity (0.1%) included 4-nonylphenol and octylphenol (≤0.1%). Notably, the cross-reactivity of structural analogues evaluated using FPIA and indirect ELISA varied marginally, with some discrepancy observed between the two methods. Similar observations have been reported previously [27], and authors of [21] also identified varying cross-reactivities using FPIA and indirect ELISA. We postulate that this behavior could be explained by binding kinetics [18]. 

### 3.4. Investigation of Matrix Effects in ELISA and FPIA

Matrix components in immunoassays can interfere with assay performance [15]. In this study, the effects of different matrix components were evaluated using ELISA and FPIA. The ELISA results showed reduced signal intensity in the presence of certain matrices, but sensitivity remained high. Dilution of samples helped mitigate the matrix effect. FPIA exhibited enhanced signal intensity and sensitivity due to the buffering capacity of the working buffer. Dilution was effective in reducing matrix interferences. Understanding and addressing the matrix effect are crucial for optimizing immunoassays. Results demonstrated in SI (Chapter S7, Figures S12 and S13, Tables S5 and S6).

## 4. Discussion

Under optimized conditions, FPIA allowed BPA to be detected at 1.0 μg/L, working range from 2 to 155 μg/L. Cross-reactivity of various molecules with bisphenol A (BPA) was evaluated in this study. Demethylation and removal of hydroxyl groups from BPA resulted in reduced cross-reactivity for BVA, 4-CP, BPE, BPF, and BPS. However, bisphenol B (BPB) showed significantly high cross-reactivity, likely due to its structural similarity to the immunization antigen. Complex analogues such as bisphenol S (BPS) could not be detected, while simple phenolic compounds exhibited low cross-reactivity. The cross-reactivity varied slightly between FPIA and indirect ELISA, potentially due to binding kinetics.

The presence of bisphenol A in our daily lives can be attributed to the usage of polymer products. Plastic sunglasses, CDs, and phones with glass screen protectors are examples of such products that can come into contact with our skin. Rubber ducks are commonly used as bath toys by children and adults alike. When warm water is added to the bathtub, however, BPAs may be released into the water. Consequently, the objective of this study was to explore the release of bisphenol A from various plastic samples utilizing the enhanced and optimized testing systems of FPIA and ELISA.

An experimental setup was implemented to test for the release of bisphenol A (BPA) from polymer materials. The samples included compact discs (CD), sunglass frames, sunglass lenses, and an iPhone screen protector (Table 1). Validation was performed using ELISA and LC–MS/MS as reference methods, with excellent agreement between the methods. The results LC–MS/MS and ELISA reflect the progressing ban of BPA use in polymer material. However, the FPIA method was unable to detect low concentrations of BPA in the iPhone screen protector. The extracts from the polymer materials showed BPA concentrations ranging from 36 to 60 μg/L.

The FPIA and ELISA results exhibit strong agreement, indicating their close correlation. The high accuracy and precision observed in these methods suggest that they are sensitive and reliable for analyzing bisphenol A (BPA) in diverse sample types.

## 5. Conclusions

The aim of this study was to develop and compare immunoassay techniques, namely a mix-and-read homogeneous FPIA and a heterogeneous reference ELISA, for the determination of the endocrine-disrupting compound bisphenol A in polymer materials. 

An indirect ELISA was optimized, conjugates (BVA–BSA and BVA–Ahx–BSA, made from BVA, a linkable BPA mimicking) were synthesized. One of the conjugates included a C6 spacer (Ahx). MALDI-ToF measurements were used to confirm the conjugation of BVA molecules to the BSA carrier protein and a spacer derivate of it (BSA–Ahx). The precision profile of the BPA calibration curve showed an LOD of 0.05 µg/L and an IC_50_ of 0.2 µg/L. Although ELISAs are suitable tools for sensitive and fast analysis with high sample throughput, they often require long reaction times and involve multiple washing steps and incubations, making the ELISA the most time-consuming format.

FPIA is a mix-and-read homogeneous assay that does not require the immobilization of reagents. FPIA is known for its quick and straightforward procedure, typically taking only 20 to 30 min to perform. This makes it a promising technique for rapid detection of BPA contamination in various samples, offering a faster alternative to instrument-based methods that often require longer analysis times. FPIA for detecting BPA was developed using structurally different tracers, including one with a C6 spacer, which were synthesized and tested for the first time. The performance of the tracer molecules was evaluated, and the FPIA was optimized to determine the influence of tracer structure on assay sensitivity. 

FPIA displayed satisfactory precision and sensitivity with an LOD of 1.0 µg/L, which is sensitive compared to previously reported LODs. The accuracy of the method was satisfactory, indicating that it is a suitable rapid and inexpensive method for detecting BPA.

The ELISA and FPIA methods demonstrated quantification limits that were significantly lower than the current specific migration limit set for bisphenol A by the EU Commission (600 µg/L). Additionally, the high sample throughput of FPIA immunoassay highlights its simplicity and efficiency, as multiple samples can be analyzed simultaneously. These findings indicate that these immunoassay techniques hold promise for monitoring bisphenol A levels in aqueous food samples.

## Figures and Tables

**Table 1:** The analytical results of the chosen samples (*n* = 3 replicates) are presented, including the levels of BPA detected by LC–MS/MS ELISA, and LFIA. The information in brackets represents the immunoassays’ recovery rate (i.e., BPA in correlation to LC–MS/MS).

	Sample	c (BPA) ± SD (μg/L)				
		LC–MS/MS (µg/L)	FPIA (µg/L)	CV%	ELISA (µg/L)	CV%
1	Compact disc (CD)	49.5 ± 4	60 ± 3.2 (121)	6	56 ± 3 (113)	5
2	Sunglass frame	144 ± 11	121 ± 6 (84)	5	132 ± 12 (91)	9
3	Sunglass lenses	36.2 ± 2	36 ± 2.4 (99)	8	32 ± 2 (88)	11
4	iPhone screen protector	1 ± 0.3	<LOD	_	1.1 ± 0.3 (106)	15

**Table 2:** The characteristics of the two tracers’ binding to the monoclonal anti-BPA antibody (mAb) (tracer dilution: 1:10,000 in borate buffer).

Tracer	Optimal Dilution		Concentration of mAb (µg/mL)	mP Value of Free Tracer (mP)	Optimal Incubation Time (s)	
	Tracer	mAb			Tracer	mAb
BVA–AMF	1:4000	1:800	8.75	114	120	90
BVA–Ahx–AMF	1:6500	1:4000	1.75	221	200	90

## Data Availability

The data presented in this study are available upon request from the corresponding author.

## Supplementary Materials

The following supporting information can be downloaded at: https://www.mdpi.com/article/10.3390/bios13060664/s1.

## Author Statement

The research presented in this article is part of Anna Raysyan’s doctoral dissertation, which concerns immunoassay techniques that are inexpensive, adaptable, fast, and user-friendly for detecting bisphenol A, an endocrine-disrupting compound. The current article focuses specifically on developing a fluorescence polarization immunoassay (FPIA) for BPA determination.
