# Supplementary material for: BPA Endocrine Disruptor Detection at the Cutting Edge: FPIA and ELISA Immunoassays
Source: Biosensors (Basel). 2023 Jun 19;13(6):664. doi: 10.3390/bios13060664 (PMC10296492; doi:10.3390/bios13060664)
Supplement: Supplementary file 1 [file biosensors-13-00664-s001.zip › biosensors-2343971-supplementary.pdf]

biosensors

Supplementary Information

BPA Endocrine Disruptor Detection at the Cutting Edge: FPIA and ELISA Immunoassays

Anna Raysyan [1,2], Sandro D. Zwigart [3], Sergei A. Eremin [4] and Rudolf J. Schneider [1,3,*]

[1] BAM Federal Institute for Materials Research and Testing, 12205 Berlin, Germany
[2] Department of Chemistry, Humboldt-Universität zu Berlin, 12489 Berlin, Germany
[3] Faculty III Process Sciences, Technische Universität Berlin, 10623 Berlin, Germany
[4] Chemical Faculty, M.V. Lomonosov Moscow State University, Moscow 119991, Russia
* Correspondence: rudolf.schneider@bam.de; Tel.: +49-30-8104-1151

1) Chemical structures of the synthesized hapten-protein conjugates and hapten-fluorophore tracers

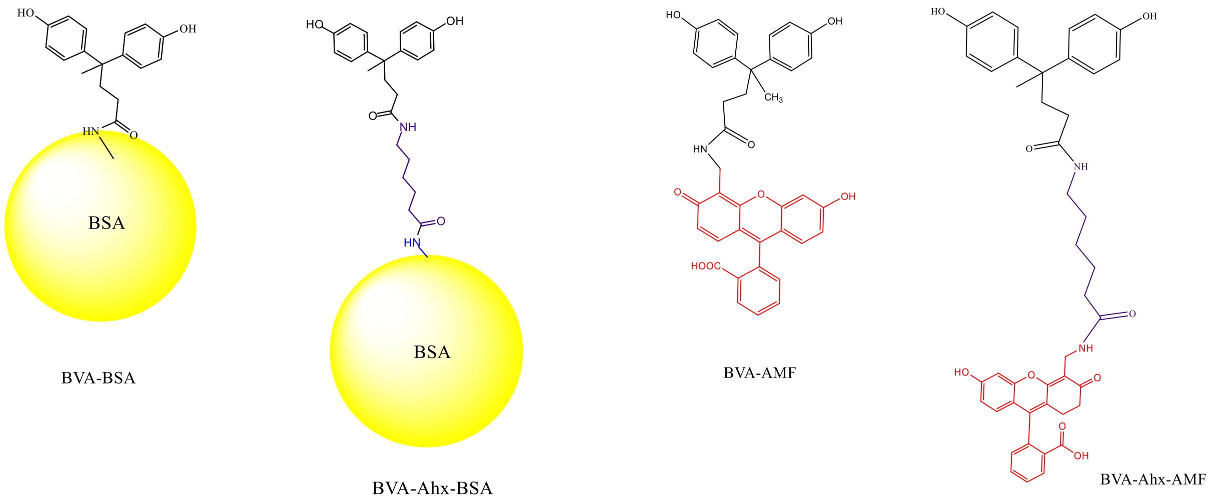

Figure S1 Chemical structures of the synthesized hapten-protein conjugates (BVA-BSA and BVA-Ahx-BSA) and hapten-fluorophore tracers (BVA-AMF and BVA-Ahx-AMF)

2) Antibody dilution curve for FPIA

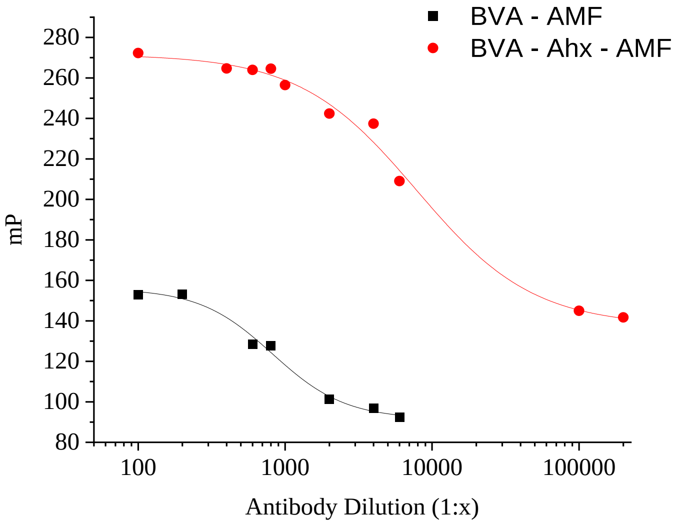

FIGURE S2 Antibody titration curves in the FPIA with the two tracers

3) Characterization of hapten-protein conjugates

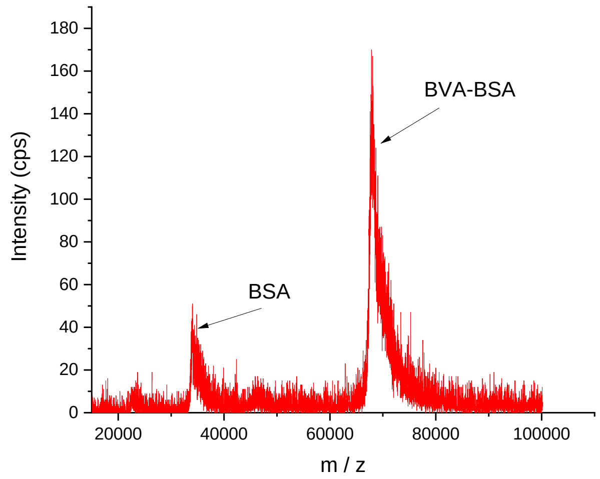

Figure S3 MALDI-TOF/MS spectra of unconjugated BSA (present in the product) and BSA conjugated with BVA. It can be seen that the mean in the mass signal distribution of the conjugate has increased significantly compared to BSA. The spectrum represents the average of three measurement (n = 3).

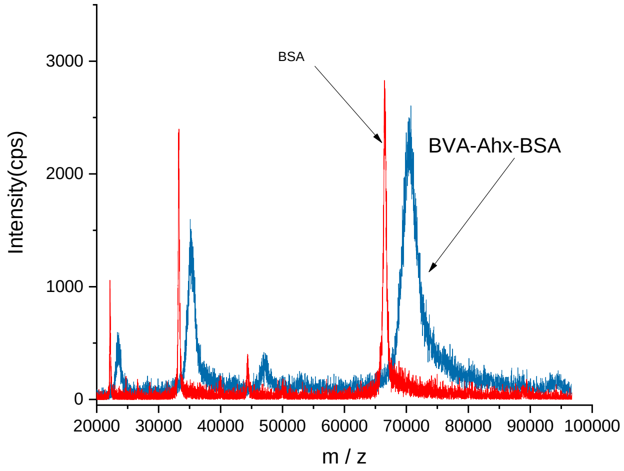

Figure S4 MALDI-TOF/MS spectra of unconjugated BSA and BSA conjugated with BVA-Ahx (blue). It can be seen that the mean in the mass signal distribution of the conjugate has increased significantly compared to BSA. The spectrum represents the average of three measurements ($n = 3$).

4) Verification of the reaction of BVA with Ahx and formation of BVA-Ahx

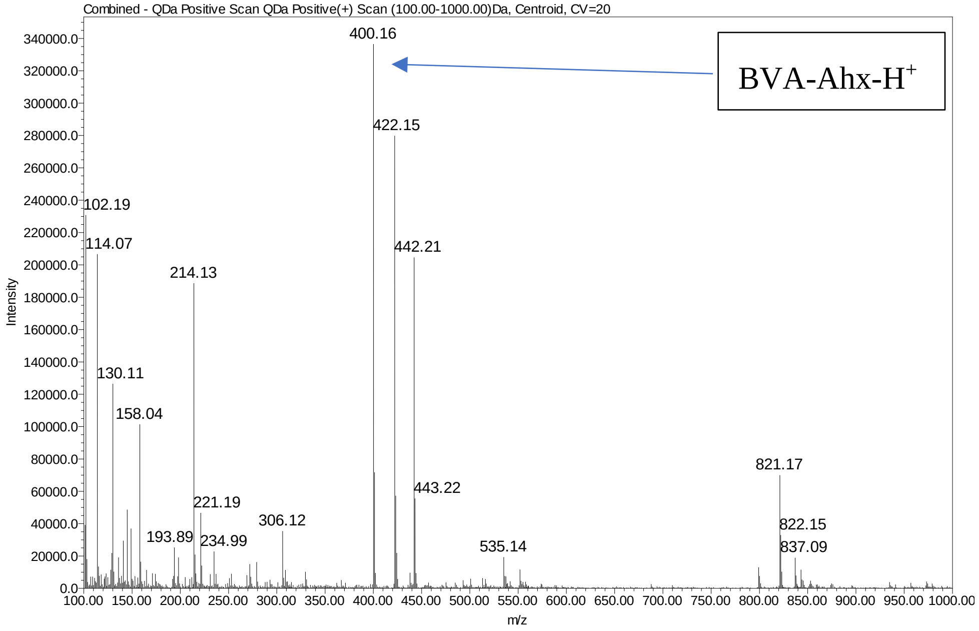

Figure S5 Mass spectrum of the largest chromatographic peak in the product of the reaction of BVA with Ahx with the largest signal at m/z (BVA-Ahx-H^+) = 400.2, which reflects the mass of the desired product (399 g/mol) plus the mass of a proton (+1).

Table S1 R_f values of the collected bands of the tracers during purification by TLC (each tracer was cleaned up for 3 times by TLC).

**Tracer**	**BVA-AMF**	**BVA-Ahx-AMF**
**TLC retardation factor R_f**	**A** 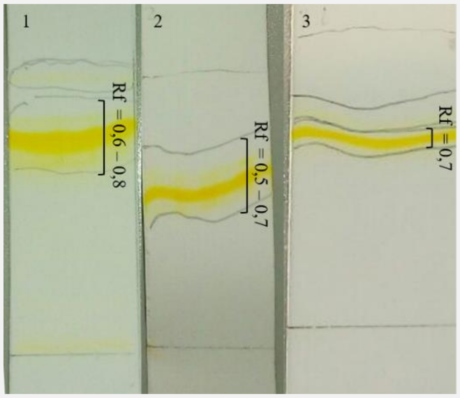	**B** 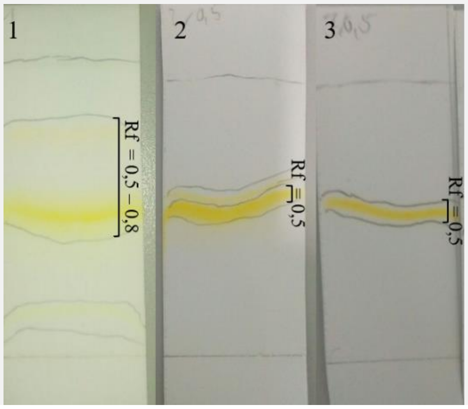

Table S2. The calibration curves' 4-parameter fitting parameters

	**A1**	**A2**	**IC_{50} μg/L**	**R^2**	**LOD μg/L**
BVA–BSA	1.09	0.02	0.4	0.99	0.08
BSA–Ahx–BSA	0.9	0.04	0.2	0.99	0.05

Table S3. Parameters of the 4-parameter fitting in the developed FPIAs using different tracers.

	**A1**	**A2**	**IC_{50} μg/L**	**R^2**	**LOD μg/L**
BVA–AMF	1.0	0.4	33	0.99	11
BSA–Ahx–AMF	1.0	0.5	7.5	0.99	1.0

Table S4. Cross-reactivity (CR, in %) of the antibody, determined by ELISA and FPIA.

Abbr.	Chemical structure	ELISA	FPIA	Abbr.	Chemical structure	ELISA	FPIA
**BPA**	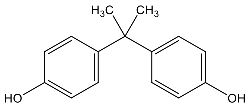	100	100	**BPF**	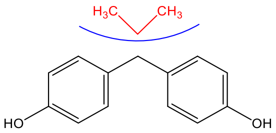	1.0	< 0.1
**BPA-d16**	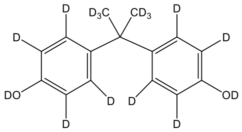	60	252	**BPS**	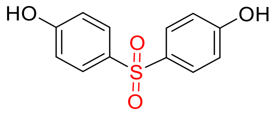	0.13	< 0.1
**BVA**	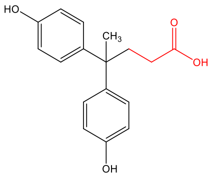	13.3	106	**BPB**	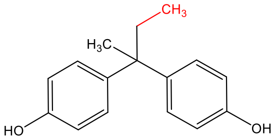	217	243
**4-CP**	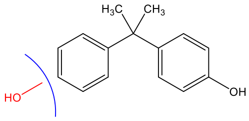	1.6	27.2	**OCP**	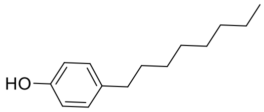	0.01	< 0.1
**BPE**	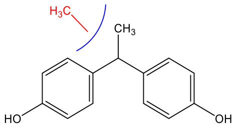	1.0	11.6	**4-NP**	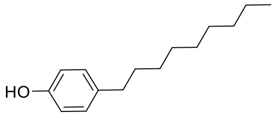	0.02	< 0.1

BPA reference concentrations of samples were determined by LC-MS/MS using an Agilent 1260 Infinity LC system with a binary pump, degasser, autosampler, and column heater with LOD 0.2 ng/mL.

The chromato-graphic separation was carried out on a Kinetex XBC18, 100 Å, 2.6 µm, 150 × 3 mm analytical LC column with an UHPLC C18, 3 mm guard column (both Phenomenex, Aschaffenburg, Ger- many). As mobile phases, Milli-Q water with 10 mM NH4Ac and 0.1 % (v/v) acetic acid (A) and MeOH with 10 mM NH4Ac and 0.1 % (v/v) AcOH (B) were used. The system was run at a flow rate of 350 µL min−1 and a column heater temperature of 30 °C. An elution gradient was applied, starting with 80 % A for the first 15 min. Within 5 min, A is decreased to 5 % (95 % B). Then, A is ramped up back to 80 % within 0.5 min and maintained at this level for 14.5 min to re-equilibrate the column. Fifteen microliters of sample were injected. Mass spectrometric detection was per- formed on an ABSciex 6500 Triple Quad mass spectrometer. Electrospray ionization (ESI) in pos- itive ionization mode was employed.

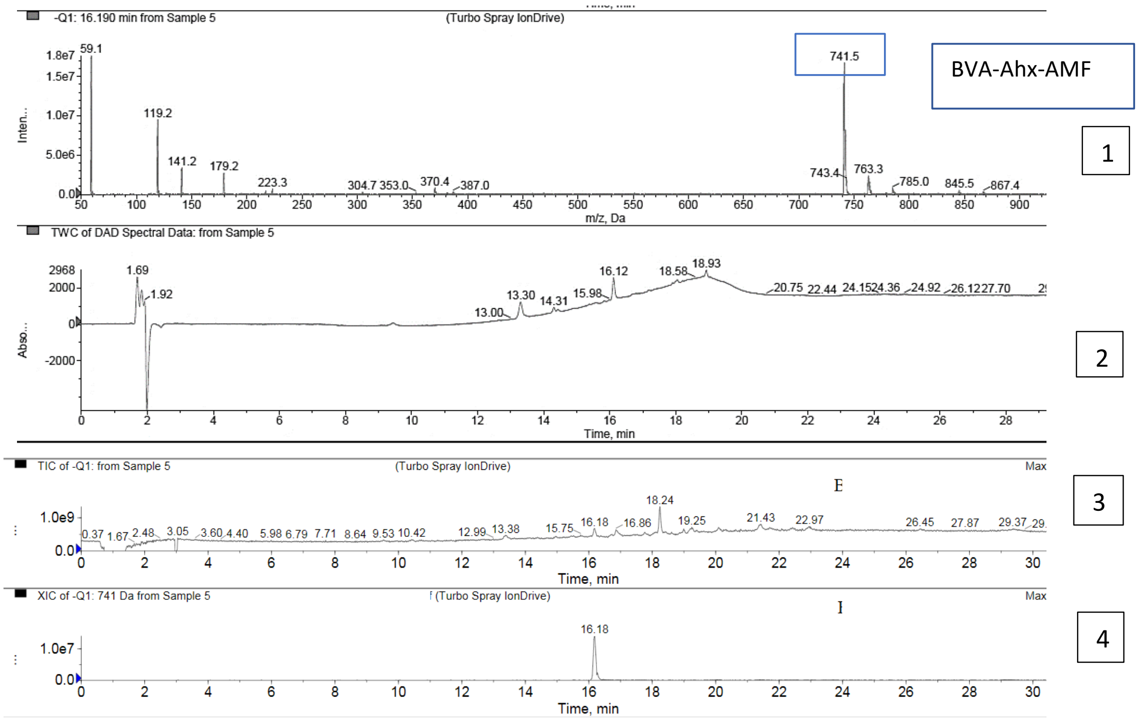

Figure S6 Synthesis of BVA-Ahx-AMF after TLC purification: (1) Mass spectrum of chromatographic peak at RT = 16.190 min, with the largest signal at m/z = 741.5 Da. This reflects the mass of the desired product (742.3 g/mol) minus the mass of a proton (-1). (2) HPLC chromatogram, DAD trace, showing larger signals at RT = 16.12 min. (3) TIC (total ion chromatogram) showing the same peaks as the DAD plus a major peak at RT = 18.24 min. (4) Extracted Ion Chromatogram (XIC, m/z = 741) showing the peak of the desired product at 16.18 min.

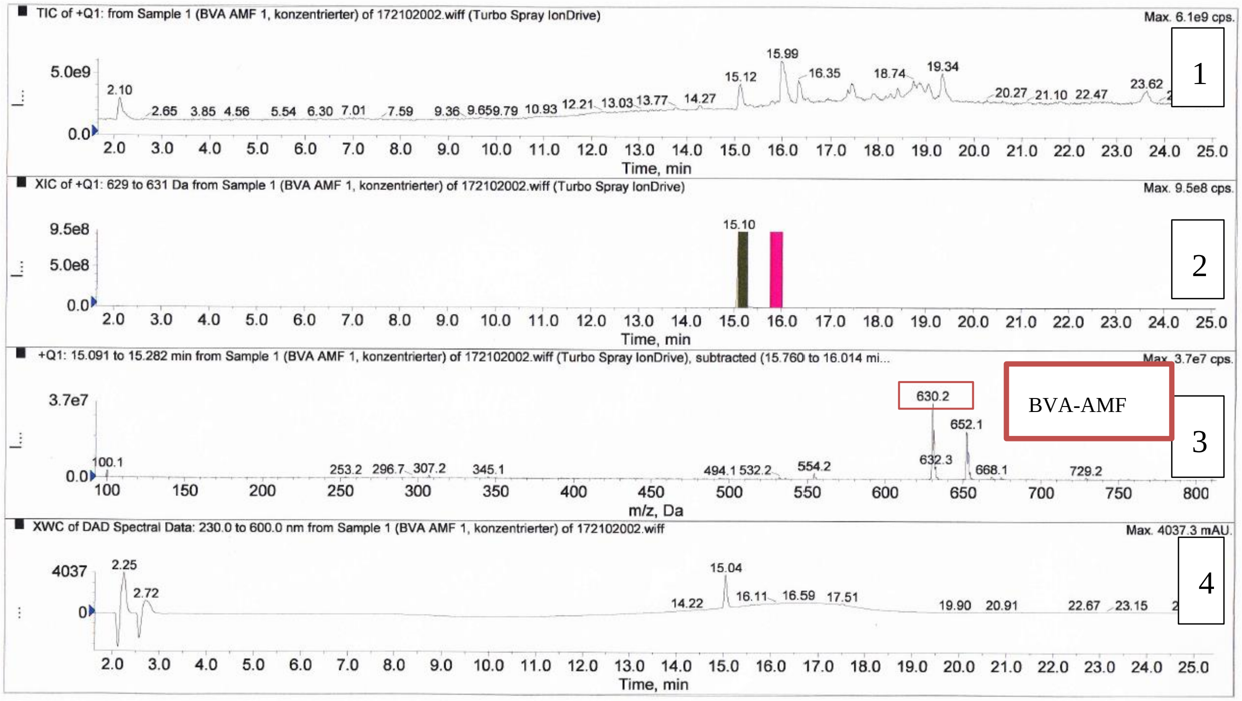

Figure S7 Synthesis of BVA–AMF. Characterization of the product before TLC purification: (1) TIC (total ion chromatogram) showing the same peaks as the DAD (trace 4) plus a major peak at RT = 15.12 min. (2) Extracted Ion Chromatogram (XIC, m/z = from 629 to 631) showing the peak of the desired product at 15.10 min. (3) Mass spectrum of the chromatographic peak at RT from 15.091 to 15.282 min, with the largest signal at m/z = 630.2 Da. This reflects the mass of the desired product (629.3 g/mol) plus the mass of a proton (+1). (4) HPLC chromatogram, DAD trace, showing the largest signal at RT = 15.04.

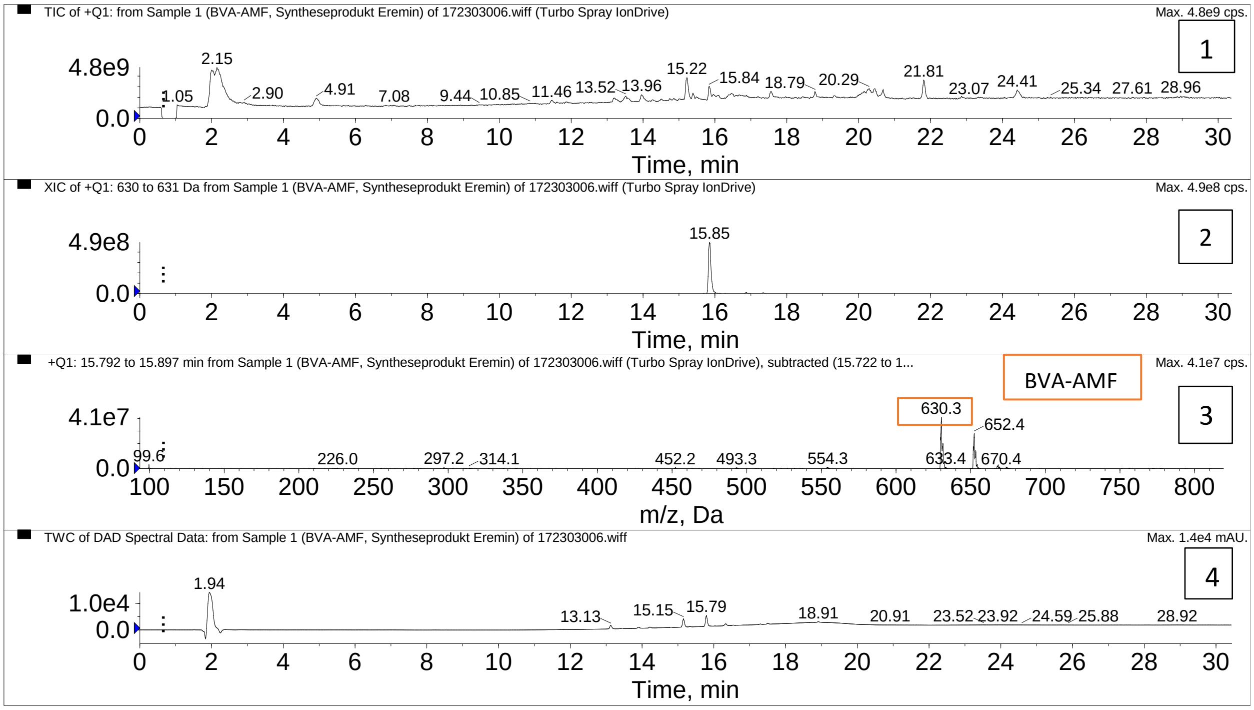

Figure S8 Synthesis of BVA–AMF. Characterization of the product after TLC purification: (1) TIC (total ion chromatogram) showing the same peaks as the DAD (trace 4) plus a major peak at RT = 15.22 min. (2) Extracted Ion Chromatogram (XIC, m/z = from 630 to 631) showing the peak of the desired product at 15.85 min. (3) Mass spectrum of chromatographic peak at RT from 15.792 to 15.897 min, with the largest signal at m/z = 630.3 Da. This reflects the mass of the desired product (629.3 g/mol) plus the mass of a proton (+1). (4) HPLC chromatogram, DAD trace, showing a signal at RT = 15.15.

6) Chromatograms to detect BPA

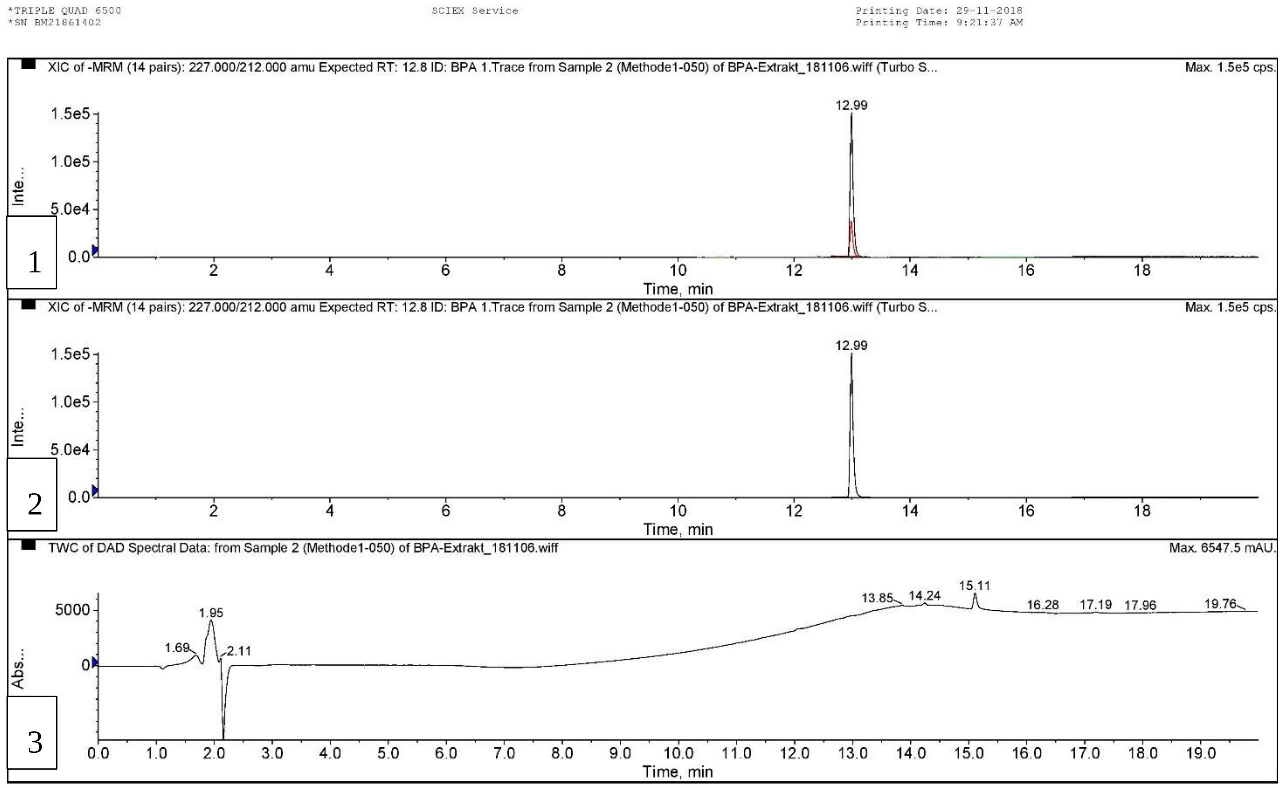

Figure S9 (Sample 1) Chromatograms of BPA (1) XIC (extracted ion chromatogram) showing the peak of the desired BPA at 12.99.(2) XIC (extracted ion chromatogram) showing the peak of the desired BPA at 12.99. (3) HPLC chromatogram, DAD trace, showing a signal at RT = 12.56.

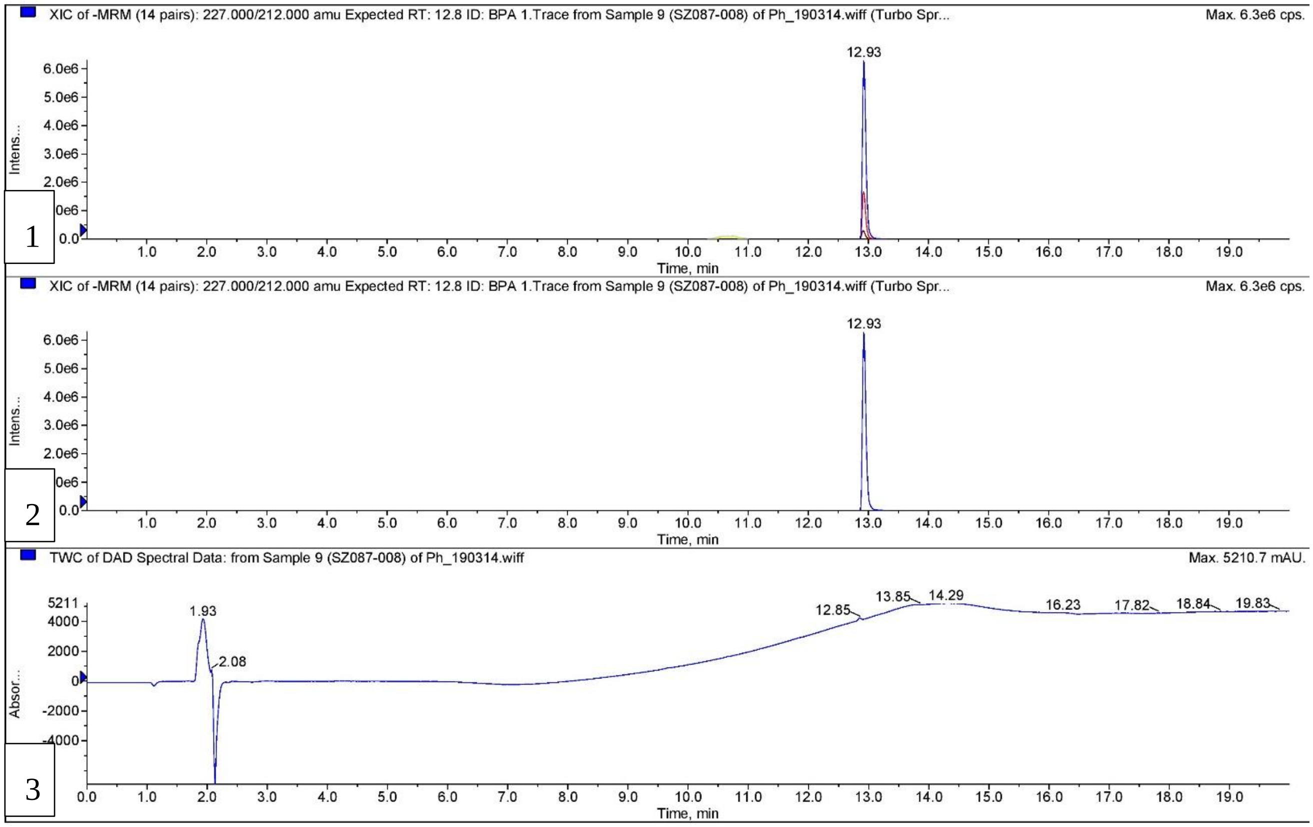

Figure S10 (Sample 2) Chromatograms of BPA (1) XIC (extracted ion chromatogram) showing the peak of the desired BPA at 12.93. (2) XIC (extracted ion chromatogram) showing the peak of the desired BPA at 12.93. (3) HPLC chromatogram, DAD trace, showing a signal at RT = 12.56.

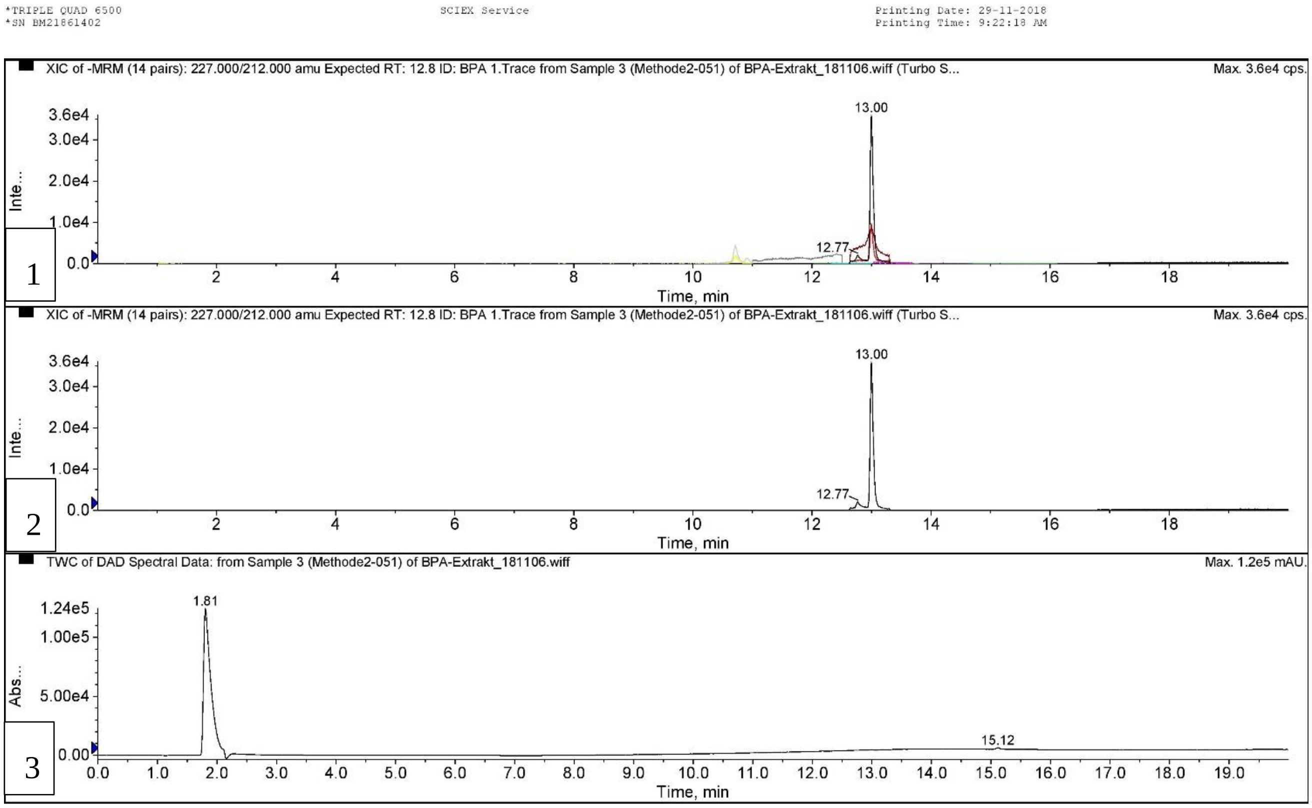

Figure S11 (Sample 3) Chromatograms of BPA (1) XIC (extracted ion chromatogram) showing the peak of the desired BPA at 13.00. (2) XIC (extracted ion chromatogram) showing the peak of the desired BPA at 13.00. (3) HPLC chromatogram, DAD trace, showing a signal at RT = 12.56.

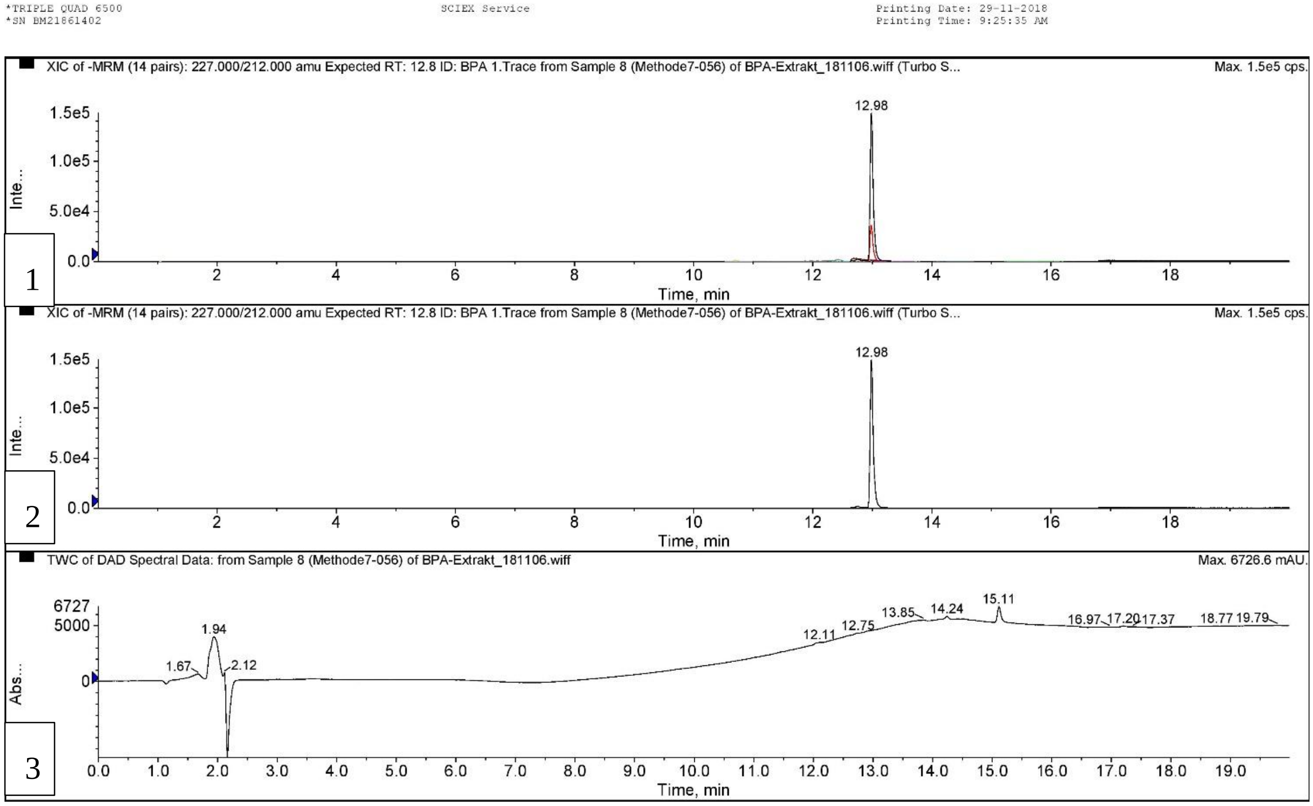

Figure S12 (Sample 4) Chromatograms of BPA (1) XIC (extracted ion chromatogram) showing the peak of the desired BPA at 12.98. (2) XIC (extracted ion chromatogram) showing the peak of the desired BPA at 12.98. (3) HPLC chromatogram, DAD trace, showing a signal at RT = 12.56.

7) The optimization of immunoassays involves evaluating the influence of matrix components, such as dissolved salts, organic solvents, and endogenous disturbances, on the assay performance. This "matrix effect" can impact the sensitivity and reliability of the immunoassay, leading to reduced accuracy. To assess interferences, a standard curve generated in a blank matrix is compared to a calibration curve in the sample matrix. If the curves overlap, the matrix effect is deemed insignificant, and the standard curve can be used for sample analysis.

In this study, the effects of various matrix components (acetone, methanol, sodium chloride, and hard water with different degrees of German hardness) were investigated using ELISA and FPIA. The ELISA results showed reduced signal intensity (A1) in the presence of certain matrices, although the sensitivity (IC50) remained relatively high. Dilution of the samples could potentially mitigate the matrix effect. Interestingly, the ELISA signal and sensitivity improved when the calcium concentration was increased, suggesting that a high-salinity buffer could minimize the matrix effect of calcium. In contrast, the FPIA results demonstrated enhanced signal intensity and

sensitivity in the presence of the matrix, likely due to the buffering capacity of the working buffer used (BB). Diluting the samples multiple times was an effective strategy to mitigate matrix interferences in FPIA and avoid false negative results.

Overall, understanding and mitigating the matrix effect are crucial for optimizing immunoassays, and strategies such as buffer selection and sample dilution can be employed to minimize the impact of matrix components on assay performance.

Table S5. Parameters of FPIA detection of BPA in different matrix.

	**A1**	**A2**	**IC_{50}**	***B* (Slope)**	**R^2**
Reference curve (STD)	1.0	0.4	7.52	0.69	0.99
Ca^{2+} 7 °dH	0.93	0.4	0.48	0.78	0.99
Ca^{2+} 14 °dH	0.94	0.4	1.6	1.1	0.99
Ca^{2+} 21 °dH	1.1	0.5	1.1	1.1	0.99
NaCl 3.5 %	0.9	0.2	1.1	1.2	0.99
Acetone 5 %	0.8	0.2	0.4	0.57	0.99
MeOH 5 %	1.0	0.3	1.3	0.54	0.99
MeOH 10 %	0.93	0.3	5.3	0.6	0.99

Table S6 Parameters of ELISA for BPA in different matrices

	**A1**	**A2**	**IC_{50}**	***B* (Slope)**	**R^2**
Reference curve (STD)	0.96	0.05	0.2	0.99	0.99
Ca^{2+} 21 °dH	0.8	0.04	0.02	0.87	0.98

Ca^{2+} 14 °dH	1.01	0.04	0.06	0.7	0.99
Ca^{2+} 7 °dH	0.68	0.05	0.02	0.97	0.99
NaCl 3.5 %	0.11	0.04	0.03	0.84	0.99
Acetone 5 %	0.6	0.04	0.1	0.88	0.98
MeOH 5 %	0.3	0.05	0.05	0.87	0.99
MeOH 10 %	0.3	0.05	0.14	0.97	0.98

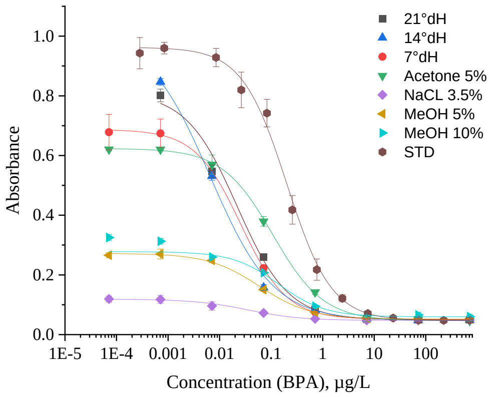

Figure S13 ELISA standard curves for BPA detection using different matrices (n = 3)

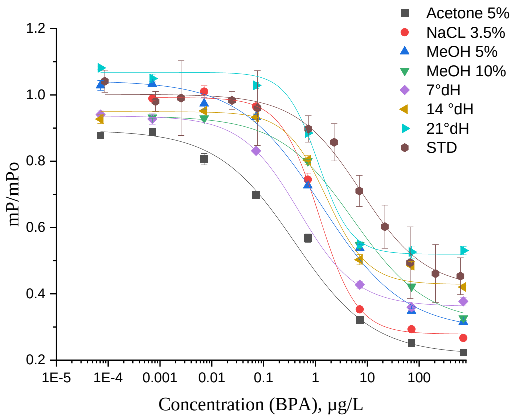

Figure S14 FPIA standard curves for BPA detection using different matrices (n = 3)
